# Correlation Among Behavior, Personality, and Electroencephalography Revealed by a Simulated Driving Experiment

**DOI:** 10.3389/fpsyg.2019.01524

**Published:** 2019-07-03

**Authors:** Lirong Yan, Yi Wang, Changhao Ding, Mutian Liu, Fuwu Yan, Konghui Guo

**Affiliations:** ^1^Hubei Key Laboratory of Advanced Technology for Automotive Components, Wuhan University of Technology, Wuhan, China; ^2^Hubei Collaborative Innovation Center for Automotive Components Technology, Wuhan, China; ^3^State Key Laboratory of Automotive Simulation and Control, Jilin University, Changchun, China

**Keywords:** personality, electroencephalography, driving behavior, source reconstruction, clustering analysis, simulated driving

## Abstract

Drivers play the most important role in the human-vehicle-environment system and driving behaviors are significantly influenced by the cognitive state of the driver and his/her personality. In this paper, we aimed to explore the correlation among driving behaviors, personality and electroencephalography (EEG) using a simulated driving experiment. A total of 36 healthy subjects participated in the study. The 64-channel EEG data and the driving data, including the real-time position of the vehicle, the rotation angle of the steering wheel and the speed were acquired simultaneously during driving. The Cattell 16 Personality Factor Questionnaire (16PF) was utilized to evaluate the personalities of subjects. Through hierarchical clustering of the 16PF personality traits, the subjects were divided into four groups, i.e., the Inapprehension group, Insensitivity group, Apprehension group and the Unreasoning group, named after their representative personality trait. Their driving performance and turning behaviors were compared and EEG preprocessing, source reconstruction and the comparisons among the four groups were performed using Statistical Parameter Mapping (SPM). The turning process of the subjects can be formulated into two steps, rotating the steering wheel toward the turning direction and entering the turn, and then rotating the steering wheel back and leaving the turn. The bilateral frontal gyrus was found to be activated when turning left and right, which might be associated with its function in attention, decision-making and executive control functions in visual-spatial and visual-motor processes. The Unreasoning group had the worst driving performance with highest rates of car collision and the most intensive driving action, which was related to a higher load of visual spatial attention and decision making, when the occipital and superior frontal areas played a very important role. Apprehension (O) and Tension (Q4) had a positive correlation, and Reasoning (B) had a negative correlation with dangerous driving behaviors. Our results demonstrated the close correlation among driving behaviors, personality and EEG and may be taken as a reference for the prediction and precaution of dangerous driving behaviors in people with specific personality traits.

## Introduction

With the increasing number of motor vehicles, the incidence of related traffic accidents is also increasing. The World Health Organization (WHO) released the Global status report on road safety in 2018 and indicated that 1.35 million people worldwide died from road traffic accidents and 50 million people were injured every year (WHO, 2018). The report of the National Bureau of Statistics of China (NBSC), indicated that in 2017, 0.203 million traffic accidents occurred in roads and 0.0638 million traffic accidents caused casualties (National Bureau of Statistics of China, 2018). Traffic accidents have become a global problem resulting in deaths, physical injuries, psychological problems and financial losses. Traffic safety research is of critical importance for individuals, families and society.

As the sensory and controlling center, humans play the most important role in the human-vehicle-environment system, and with the development of advanced driver assistance systems, humans have become the primary factor in traffic accidents (Petridou and Moustaki, 2000), accounting for 45–75% (Wierwille et al., 2002), or even up to 95% (Rumar, 1990) of road accidents. Many dangerous driving behaviors, such as drunk driving (Krüger, 2013), motor vehicle retrograde (Zhao et al., 2009), speeding (Chung and Wong, 2010), fatigue driving (Zhang et al., 2016), and distracted driving (Lansdown et al., 2015) can directly lead to accidents. Many efforts are being made to eliminate human factor related accidents worldwide such as the “Human Factors in Connected Vehicles” initiative of the National Highway Traffic Safety Administration (Lerner et al., 2014) and the “Adaptive Integrated Driver-vehicle Interface” initiative in Europe (Amditis et al., 2005).

Driving is a complex and multifaceted behavioral process, which is affected by psychological, physiological and physical factors. Ample evidence has demonstrated the influence of the cognitive state of a driver (Renner and Anderle, 2000; Lajunen, 2001) and his/her personality, on driving behavior. The relationship between personality and driving is usually explored using a questionnaire investigation. According to Eysenck’s Personality Questionnaire (EPQ, classifying personality as extraversion, neuroticism, psychoticism) (Eysenck and Eysenck, 1965) investigation, an extroverted personality was positively correlated with traffic accidents (Lajunen, 2001), driving error (Ben-Ari et al., 2016) and illegal behavior (Guo et al., 2016). Neuroticism was associated with aggressive, offensive driving (Jovanović et al., 2011), and was more likely to induce driving fatigue (Šeibokaité et al., 2014) and risky driving behaviors (Booth-Kewley and Vickers, 1994). Psychoticism was found to significantly correlate with driving skills (Alavi et al., 2017), but not significantly with driving accidents (Renner and Anderle, 2000). According to the five factor model (FFM, classifying the personality as Neuroticism, Extraversion, Openness, Agreeableness and Conscientiousness) (Digman, 1990) investigation, neuroticism and extraversion were positively correlated with risky driving (Mallia et al., 2015) and aggressive driving (Dahlen and White, 2006), the personality traits of conscientiousness and agreeableness were negatively correlated with risky driving (Cellar et al., 2000). Openness was reported to be the best predictors of aggressive driving (Mallia et al., 2015). Many researchers utilized the 16 Personality Factor Questionnaire (16PF) (Zhang et al., 2009; Manglam et al., 2013) to explore the relationship between drivers’ personality traits and driving. The 16PF is a comprehensive measurement of normal adult personality in terms of the 16 personality dimensions, classifying personality as Warmth (A), Reasoning (B), Emotional Stability (C), Dominance (E), Liveliness (F), Rule-Consciousness (G), Social Boldness (H), Sensitivity (I), Vigilance (L), Abstractedness (M), Privateness (N), Apprehension (O), Openness to Change (Q1), Self-Reliance (Q2), Perfectionism (Q3), and Tension (Q4). There were significant differences in personality traits between drivers with no accident history and accident-prone drivers or chronic violators. Sensitivity (I), Tension (Q4), and Perfectionism (Q3) were related to safe driving, and Openness to Change (Q1) and Abstractedness (M) were related to dangerous driving behavior (Suhr, 1953; Brown, 1976; Hilakivi et al., 1989; Manglam et al., 2013). Drivers with higher scores in Emotional Stability (C), Liveliness (F), Warmth (A), Social-boldness (H) and Dominance (E) and lower scores in Vigilance (L), Apprehension (O), and Self-Reliance (Q2), had a higher accident incidence (Zhang et al., 2009).

Besides personality, the cognitive state greatly and directly affects driving behavior. Many researches indicated the influence of the cognitive state on driving such as the attentional state (alertness, distraction, fatigue) and the emotional state (depression, anxiety, compulsion). Fatigue driving would impair the drivers’ physical characteristics, such as heart rate, time deviation of speed anticipation, systolic blood pressure, time for dark adaption, eyesight, dynamic visual acuity, reaction time to sound and reaction time to light (Zhang et al., 2014). Anxiety would ingest the cognitive resources of drivers (Eysenck and Byrne, 1992) and cause an augmented reporting of dangerous driving behaviors (Dula et al., 2010). Depression may also affect driving skills and behaviors (Nnjjm et al., 2017) and its severity was positively correlated with a standard deviation of the lateral position (Wingen et al., 2006). Traditionally, the cognitive state was measured by questionnaires such as the Fatigue Assessment Scale (Michielsen et al., 2003), the Hamilton Anxiety Scale (Maier et al., 1988) and the Hamilton Depression Rating Scale (Williams, 1988). Recently, with the development of the physiological and psychological perception techniques, the cognitive state of subjects can be measured in a more objective and quantitative manner. Among these techniques, electroencephalography (EEG) is a reliable and significant method of measuring neurophysiological activity in the human brain and the psychological state of drivers when driving. Using advanced data mining techniques, the EEG signal can be utilized to identify a driver’s alertness (Chuang et al., 2015), to predict the distraction (Wang et al., 2015), to study a driver’s perception of signal lights (Wang et al., 2008), to monitor a driver’s driving states (Peng and Wu, 2009), and to predict a driver’s intention to emergency brake (Kim et al., 2014).

Currently, the potential correlation of cognitive function and personality and its effect on driving behavior is complicated and remains unclear. In this paper, we tried to explore the correlation between driving behavior, personality and EEG using a simulated driving task and the corresponding data analysis. Thirty-six healthy subjects participated in the study. The 64-channel EEG data and the driving data, including the real-time position of the vehicle, the rotation angle of the steering wheel and the speed were acquired simultaneously during driving. The Cattell 16 Personality Factor Questionnaire (16PF) was utilized to evaluate the personalities of subjects. Through hierarchical clustering of the 16PF personality traits, subjects were divided into four groups. The EEG difference and driving behaviors between the four groups were compared. The results indicated a correlation between driving behavior, personality traits and EEG, which might be helpful to improve the integrated human-vehicle-environment model as well as traffic safety.

## Materials and Methods

### Method Overview

**FIGURE 1:**
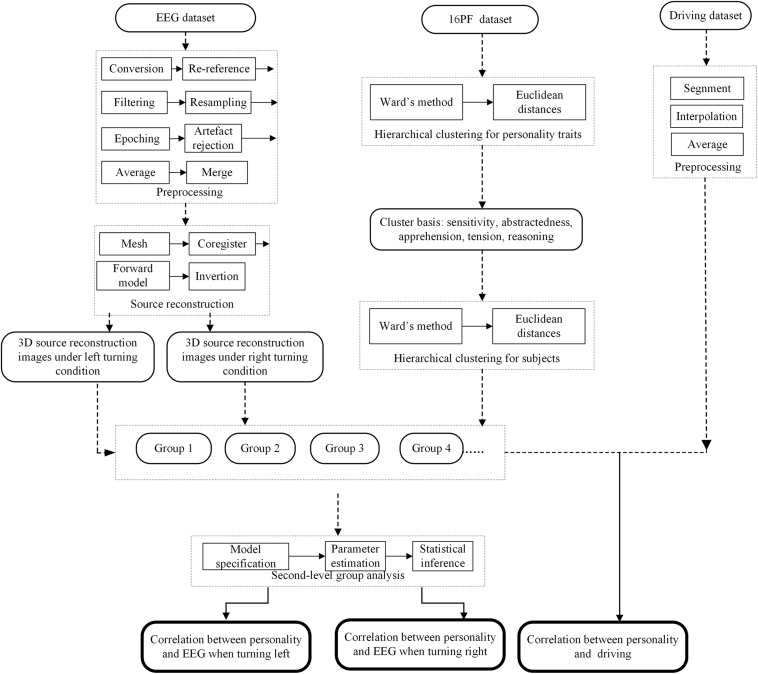
Flow diagrams showing the processing steps for correlation analysis. The flow in the dashed line indicates the preprocessing procedure, the flow in black indicates the procedure of correlation analysis, and bold frames indicate output.

The processing schema is shown in Figure 1. The following steps were included: (i) clustering analysis, to classify subjects into different groups according to their personality traits; (ii) preprocessing of EEG data and driving data; (iii) driving data analysis; (iv) EEG source reconstruction; (v) the second level group analysis, to explore the correlation between driving behavior, personality and EEG.

### Subjects and Experiment Design

Thirty-six healthy subjects (21–46 years old, mean age 27.0 ± 7.8 years, driving years: 5.2 ± 8.4 years, 27 males and nine females) were recruited. All subjects have a driving license and have real driving experience, driving in their daily life. Subjects reported no neurological or psychiatric problems and were all right-handed. Written informed consent was provided by all subjects and the data were anonymized. The study was approved by the ethical review committee of the Wuhan University of Technology.

**FIGURE 2:**
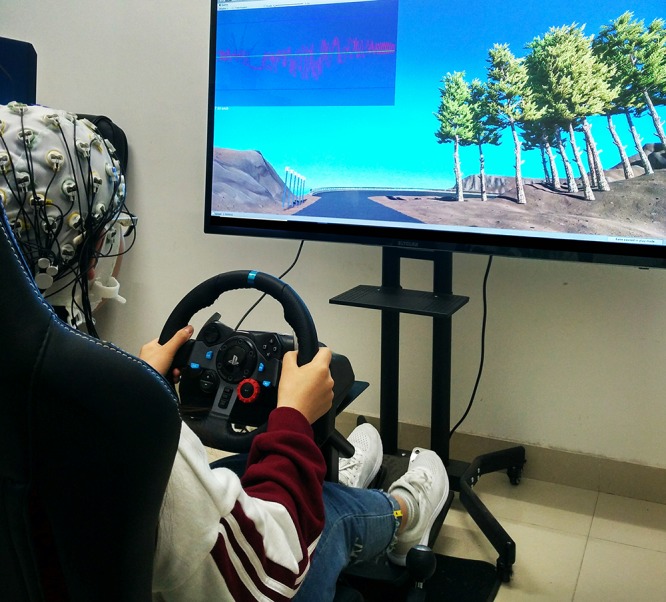
Simulated driving platform. The subject has provided written consent for the publication of this image.

Subjects were instructed to sit comfortably wearing EEG caps and to drive on a driving simulator platform (Figure 2). The platform consisted of a driving simulator (G29, Logitech, Switzerland) and a screen. The Logitech playseat consisted of a highly simulated steering wheel, a full-size driving seat, gears, accelerator and brakes. Unity 3D software (Unity Technologies, America) was employed to design the simulated driving scenario, which consisted of a 7 km circular runway with three left and four right turns. The subjects were instructed to keep their attention on driving and completed two to four driving sessions with a speed limit of 70 km/h. Each session contained four rounds and was accomplished in approximately 7 min. After each session the subjects took a break for a few minutes to avoid driving fatigue. Each subject completed three sessions. The actions of the left and right turning were marked as events when the driver noticed the roadside direction board at the beginning of the curve and made the specific actions. We videotaped the subject’s driving behavior simultaneously. Errors including driving out of the road and car collisions were recorded by the researchers.

### Data Acquisition

The driving data, including the real-time position of the vehicle, the rotation angle of the steering wheel and the speed, were acquired using C# scripts based on Unity 3D. Subjects’ brain activities were collected at 1000 Hz using the actiCHamp Amplifier (Brain Products GambH, Gilching, Germany) with 64 surface Ag/AgCl electrodes fixed on a recording cap, consistent with the international 10–20 system referenced to the Fz electrode during the driving experiment. All the subjects filled the 16PF questionnaire in after the driving experiment.

### Clustering of 16PF Scores and Subject Grouping

In 16PF, all personality traits are evaluated using a score from 1 (low) to 10 (high), where 3 and below are considered low scores, while eight and above are considered high scores. The 36 subjects were divided into different groups according to their personality traits using the agglomerative hierarchical clustering algorithm (SPSS 22.0, IBM, United States). Hierarchical clustering seeks to form a hierarchy of clusters, either by a “bottom up” agglomerative approach (the clusters would merge if their Euclidean distances were small) or by a “top down” divisive approach (a cluster would split if its scope was too large) (Rokach and Maimon, 2005). First, the 16 personality traits were divided into several categories using Euclidean distances and Ward’s method. Then the most representative personality traits were picked out, based on which the subjects were hierarchically clustered into different groups. We utilized the least-significant difference method (Atkinson, 2002) for multiple comparisons between groups to explore the relationship of the selected personality traits and aberrant driving behaviors between groups.

### Analysis of the Driving Data

The steering wheel angle data with a peri-stimulus window of 0–10 s for all left and right turns of all the subjects were extracted. The relative increment of the steering wheel angle to the first angle at time 0 were calculated and the mean curves of each group of subjects under left and right turning conditions were then obtained. The least square estimate was performed to estimate the slope of two segments of the curves as an angular velocity for each group. Their characteristics were analyzed.

### Analysis of EEG Data

The EEG signals were preprocessed with MATLAB (R2018a, MathWorks, American) and SPM12.^1^ The preprocessed process included conversion, montage, filter, downsample, epoch, merge, removing artifacts and averaging. First, the raw EEG data were converted to the format available for Statistical Parameter Mapping (SPM). Then all channels of the data were re-referenced by subtracting from the reference channel (Fz). Next, the EEG signals were band-pass filtered in the range of 0.1–30 Hz, to selectively eliminate noise and down sampled to 200 Hz to reduce the sample size. Then, the EEG epochs with a peri-stimulus window of −100 to 1000 ms were extracted. Time 0 denoted the moment the subjects began to turn, which was determined by the time that the vehicle passed by the direction board. The artifacts were removed with the threshold for eye movements or muscular activity exceeding 100 μV. The threshold was set at 0.2 for the bad channel, which would be excluded in the processing which followed. Robust averaging was performed to produce an event related potential (ERP) under two driving conditions (turning left and turning right), respectively.

The ERPs were utilized for source reconstruction, which was conducted to project 2D sensor data into a 3D brain space, to locate the exact anatomical structures of the brain activity (Litvak et al., 2011). Source space modeling, data co-registration, forward computation using the Boundary Element Method (BEM) (Jatoi et al., 2015), and inverse reconstruction using the Multiple Sparse Priors (MSP) algorithm, were performed. The time window of inversion was set as −100 to 1000 ms, which was based on an empirical Bayesian approach. Finally, 3D images containing root mean square (RMS, unsigned) source estimates corresponding to two driving conditions (turning left and turning right) for each subject were obtained and then compared between the different groups using one-way analysis of variance (ANOVA, *P* < 0.05, family wise error (FWE) correction, extent threshold *k* > 70). Age, driving years and gender were utilized as the covariates.

## Results

### Personality Traits and Clustering Results

**FIGURE 3:**
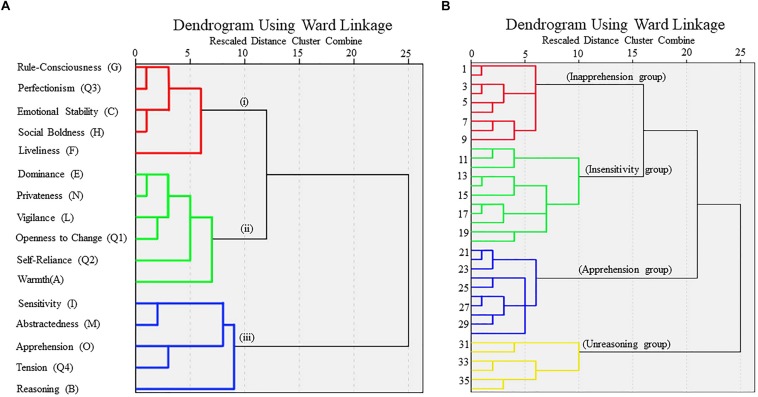
Dendrograms from Hierarchical Clustering (Ward’s method, Euclidean distance, SPSS 22.0). **(A)** Dendrogram from Hierarchical Clustering of Cattell 16 personality factors. **(B)** Dendrograms from Hierarchical Clustering of the subjects based on five personality traits [Sensitivity (I), Abstractedness (M), Apprehension (O), Tension (Q4), Reasoning (B)]. 36 subjects were clustered into four groups, from top to bottom, Inapprehension group, Insensitivity group, Apprehension group, and Unreasoning group.

Sixteen personality traits of all the subjects were all within the normal range and they were divided into three clusters (Figure 3A), which were (i) Rule-Consciousness, Perfectionism, Emotional Stability, Social Boldness and Liveliness; (ii) Dominance, Privateness, Vigilance, Openness to Change, Self-Reliance and Warmth; (iii) Sensitivity, Abstractedness, Apprehension, Tension and Reasoning. The Euclidean distance between cluster (ii) and (iii) was the smallest, therefore, the personality traits in these two clusters were utilized to conduct the second hierarchical clustering of the subjects. The subjects were divided into four groups according to the five personality traits in cluster (iii) (Figure 3B). Four groups had extremely significant differences in personality of Reasoning (*F* = 18.852, *P* < 0.0005), Apprehension (*F* = 21.856, *P* < 0.0005), and Sensitivity (*F* = 7.092, *P* < 0.001). Four groups had significant differences in personality of Emotional Stability (*F* = 4.203, *P* = 0.013), Dominance (*F* = 2.934, *P* = 0.048), Abstractedness (*F* = 3.554, *P* = 0.025), Perfectionism (*F* = 6.144, *P* = 0.002), and Tension (*F* = 3.424, *P* = 0.029, Table 1). The subjects were also divided the into four groups according to the six personality traits in cluster (ii), but the ANOVA analysis revealed no significant difference between these groups. Accordingly, the subjects were grouped based on personality traits in cluster (iii). The pairwise comparison was conducted for these five personality traits between the four groups (LSD-*t* test, *P* < 0.05, Table 2). The group with significantly lower scores in Apprehension (O), Sensitivity (I), or Reasoning (B) than the other three groups was named as the Inapprehension group, Insensitivity group and Unreasoning group, respectively. The group with the highest scores in Apprehension (O) and who also had a significant difference to the Inapprehension group and Insensitivity group was named as the Apprehension group. As for the driving performance, the number of car collisions were significantly different between the four groups (ANOVA, *P* < 0.05) and the pairwise comparison indicated that the Unreasoning group had significantly more car collisions than the other three groups (LSD-*t* test,  *P* < 0.05). The number of times driving out of the road between four groups were not signifi- cantly different, but the Unreasoning group drove out of the road

**TABLE 1:** The normalized 16PF personality traits and aberrant driving behaviors of the four groups of subjects ($\bar{X}\pm s$).

		**Inapprehension**	**Insensitivity**	**Apprehension**	**Unreasoning**		
	**Total**	**group**	**group**	**group**	**group**		
**Feature**	**(*n* = 36)**	**(*n* = 9)**	**(*n* = 11)**	**(*n* = 10)**	**(*n* = 6)**	***F***	***P***
Warmth (A)	4.72±1.86	5.67±3.73	3.73±1.49	4.70±1.83	5.17±2.14	2.121	0.117
Reasoning (B)	6.17±2.20	7.67±5.82	5.82±1.66	7.30±0.67	2.67±1.86	18.852^∗∗^	<0.0005
Emotional Stability (C)	5.19±1.58	5.11±6.36	6.36±1.43	4.30±1.64	4.67±1.37	4.203^*^	0.013
Dominance (E)	4.36±1.38	4.56±4.64	4.64±0.92	3.40±0.84	5.17±1.47	2.934^*^	0.048
Liveliness (F)	5.78±1.49	6.22±5.73	5.73±1.68	5.20±1.62	6.17±0.98	0.894	0.455
Rule-Consciousness (G)	5.19±1.58	5.89±5.64	5.64±1.63	4.40±1.51	4.67±1.03	2.110	0.119
Social Boldness (H)	5.00±1.33	5.44±5.45	5.45±1.21	4.30±1.16	4.67±1.63	1.958	0.140
Sensitivity (I)	6.44±1.27	7.22±5.27	5.27±1.27	6.80±1.03	6.83±0.75	7.092^∗∗^	<0.001
Vigilance (L)	4.17±1.18	3.89±3.91	3.91±1.38	4.40±1.17	4.67±0.52	0.813	0.496
Abstractedness (M)	7.08±1.44	7.67±6.18	6.18±1.33	7.80±1.32	6.67±1.63	3.554^*^	0.025
Privateness (N)	4.42±1.36	4.67±3.91	3.91±1.51	4.50±1.27	4.83±0.98	0.798	0.504
Apprehension (O)	6.64±1.69	4.78±6.09	6.09±0.94	8.20±0.92	7.83±1.17	21.856^∗∗^	<0.0005
Openness to Change (Q1)	4.78±1.24	5.11±4.64	4.64±1.29	4.50±0.97	5.00±1.79	0.470	0.705
Self-Reliance (Q2)	4.97±1.59	5.44±4.64	4.64±1.36	5.40±2.01	4.17±1.47	1.196	0.327
Perfectionism (Q3)	5.86±1.36	6.89±6.27	6.27±1.1	4.90±0.74	5.17±1.33	6.144^∗∗^	0.002
Tension (Q4)	5.89±1.62	5.33±5.09	5.09±1.58	6.60±1.26	7.00±2.10	3.424^*^	0.029
Times of driving out of the road	5.17±6.55	3.33±3.74	4.64±3.78	3.10±3.07	8.17±5.78	2.374	0.089
Times of car collision	5.86±4.48	4.11±2.37	5.09±4.74	5.50±5.28	10.33±2.16	3.049^*^	0.043
Driving Time (s)	416.24±53.47	420.13±51.96	430.18±46.38	405.08±43.60	403.40±84.11	0.503	0.683

^*^P < 0.05, ^∗∗^*P* < 0.01.

**TABLE 2:** Multiple Comparisons of five personality traits and aberrant driving behaviors between groups.

		**Insensitivity**	**Apprehension**	**Unreasoning**
**Feature**	**Group**	**group**	**group**	**group**
Reasoning (B)	Inapprehension group	0.006^∗∗^	0.568	0.000^∗∗^
	Insensitivity group	–	0.020^*^	0.000^∗∗^
	Apprehension group	–	–	0.000^∗∗^
Sensitivity (I)	Inapprehension group	0.000^∗∗^	0.380	0.480
	Insensitivity group	–	0.002^∗∗^	0.006^∗∗^
	Apprehension group	–	–	0.951
Abstractedness (M)	Inapprehension group	0.017^*^	0.826	0.156
	Insensitivity group	–	0.008^∗∗^	0.470
	Apprehension group	–	–	0.103
Apprehension (O)	Inapprehension group	0.007^∗∗^	0.000^∗∗^	0.000^∗∗^
	Insensitivity group	–	0.000^∗∗^	0.002^∗∗^
	Apprehension group	–	–	0.489
Tension (Q4)	Inapprehension group	0.716	0.070	0.039^*^
	Insensitivity group	–	0.025^*^	0.016^*^
	Apprehension group	–	–	0.602
Times of driving out of the road	Inapprehension group	0.471	0.899	0.028^*^
	Insensitivity group	–	0.383	0.090
	Apprehension group	–	–	0.019^*^
Times of car collision	Inapprehension group	0.601	0.469	0.007^∗∗^
	Insensitivity group	–	0.822	0.017^*^
	Apprehension group	–	–	0.030^*^

LSD-*t* test. ^*^P < 0.05, ^∗∗^*P* < 0.01.

significantly more times than the Inapprehension and Apprehension group (LSD*-t* test, *P* < 0.05). The other comparisons revealed no significance. There was no significant difference in driving time between the four groups.

### Driving Features

**FIGURE 4:**
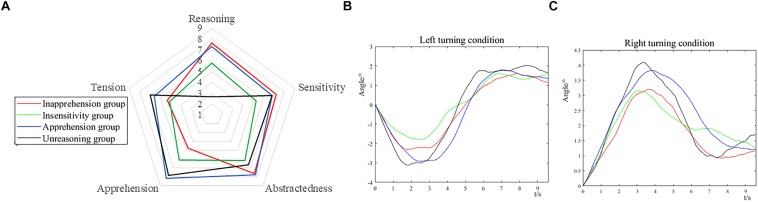
**(A)** Five selected personality traits of 16PF, averaged over subjects in each group. **(B)** The steering angle of the four groups under the left turning condition. **(C)** The steering angle of the four groups under the right turning condition. Decreasing angle corresponds to counterclockwise rotation, increasing angle corresponds to clockwise rotation.

The steering angles of four groups are shown in Figure 4 and the detailed data are listed in Table 3. There seemed to be two obvious peaks in each curve and the least square estimate was performed to estimate the slope of two segments of the curves, which represented the mean angular velocities. The turning process can be formulated in two steps, i.e., (i) rotating the steering wheel toward the turning direction, modulating the head direction and entering the turn and then (ii) rotating the steering wheel back and leaving the turn.

**TABLE 3:** Driving feature of the four groups under the left turning and right turning conditions.

**Task**	**Group**	**First step**			**Second step**			**Total time (s)**
		**Rotation**	**Angular**		**Rotation**	**Angular**		
		**angle (°)**	**velocity (°/s)**	**Time (s)**	**angle (°)**	**velocity (°/s)**	**Time (s)**	
Turning left	Inapprehension group	–2.2994	–1.3501	1.8653	1.6087	0.8264	5.9474	7.8127
	Insensitivity group	–1.7948	–0.7109	2.4376	1.6079	0.8331	4.3304	6.7680
	Apprehension group	–2.9016	–1.2506	2.3969	1.7668	1.3112	4.5966	6.9935
	Unreasoning group	–3.1045	–1.8599	1.8039	1.7705	1.4134	3.9998	5.8037
Turning right	Inapprehension group	3.1981	0.9666	3.645	0.9218	–0.6762	4.0448	7.6898
	Insensitivity group	3.1531	1.1263	3.0507	1.8565	–0.4698	3.2049	6.2556
	Apprehension group	3.8346	1.0625	3.8502	1.2045	–0.5894	5.5171	9.3673
	Unreasoning group	4.0992	1.3732	3.3372	0.9489	–0.9293	4.0661	7.4033

In the first step, under a left turning condition, the absolute angular velocity was Unreasoning group > Apprehension group > Inapprehension group > Insensitivity group; the absolute angular velocity was Unreasoning group > Inapprehension group > Apprehension group > Insensitivity group. In the second step, under the left turning condition, the absolute rotation angle was Unreasoning group > Apprehension group > Inapprehension group > Insensitivity group; the absolute angular velocity was Unreasoning group > Apprehension group > Insensitivity group > Inapprehension group.

In the first step, under the right turning condition, the absolute angular velocity was Unreasoning group > Apprehension group > Inapprehension group > Insensitivity group; the absolute angular velocity was Unreasoning group > Insensitivity group > Inapprehension group > Apprehension group. In the second step, under the right turning condition, the absolute rotation angle was Insensitivity group > Apprehension group > Unreasoning group > Inapprehension group; the absolute angular velocity was Unreasoning group > Inapprehension group > Apprehension group > Insensitivity group. Under the left turning condition, the two times needed to finish the two steps of turning were Inapprehension group > Apprehension group > Insensitivity group > Unreasoning group; under the right turning condition, the two times needed to finish the two steps of turning were Apprehension group > Inapprehension group > Unreasoning group > Insensitivity group.

### EEG Features

#### EEG Source Reconstruction Results of All Subjects

**FIGURE 5:**
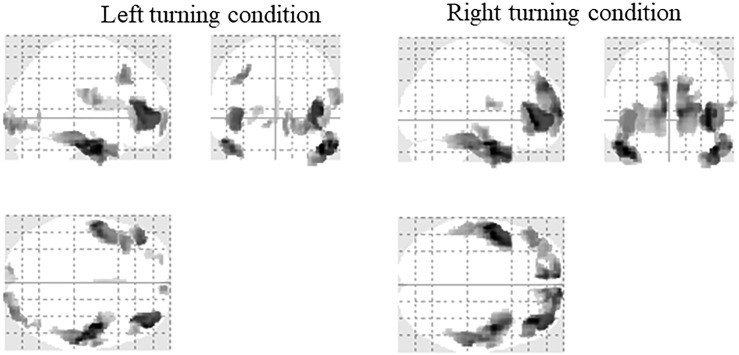
Activation of all the subjects under the two driving conditions (SPM12, ANOVA, *P* < 0.05, FWE-corrected, extent threshold *k* > 70).

Electroencephalography source reconstruction results of all the subjects under the two driving conditions are shown in Figure 5 and the details are listed in Table 4. Under the left turning condition, the bilateral temporal gyrus, frontal gyrus and the occipital gyrus were activated. Under the right turning condition, the bilateral temporal gyrus and frontal gyrus were activated. No different activation was found between the two conditions.

**TABLE 4:** Activation of all the subjects under the left turning and right turning conditions.

						**Cluster**
						**size**
**Task**	**Anatomy**	**Peak location**			***t***	**(Voxels)**
		***x***	***y***	***z***		
Turning left	Inferior temporal gyrus	46	−6	−32	7.58	827
	Middle frontal gyrus	46	46	6	7.12	733
	Middle temporal gyrus	−54	−8	−26	6.90	508
	Inferior frontal gyrus, triangle part	−40	40	−2	6.43	220
	Inferior frontal gyrus, orbital part	−40	40	−4	6.39	156
	Inferior occipital gyrus	30	−94	−12	5.89	369
	Middle frontal gyrus	−36	22	40	5.85	169
	Rolandic operculum	62	−6	14	5.55	247
	Supramarginal gyrus	60	−18	24	5.26	95
Turning right	Middle temporal gyrus	−54	−8	−26	8.48	881
	Inferior frontal gyrus, triangle part	38	40	−4	8.20	708
	Inferior frontal gyrus, triangle part	40	34	8	7.79	399
	Superior frontal gyrus, medial part	−6	52	32	7.43	984
	Superior frontal gyrus, medial part	12	66	6	7.33	528
	Middle temporal gyrus	50	−4	−26	7.09	435
	Superior frontal gyrus	18	60	6	6.74	221
	Inferior frontal gyrus, orbital part	−46	38	−10	6.18	339
	Rolandic operculum	62	−6	14	5.77	81
	Frontal gyrus, orbital part	12	62	−8	5.52	86

*SPM12, ANOVA, *P* < 0.05, FWE-corrected for the left turning and right turning, extent threshold *k* > 70. The location is in MNI coordinates.*

#### EEG Source Reconstruction Results of Four Groups

**FIGURE 6:**
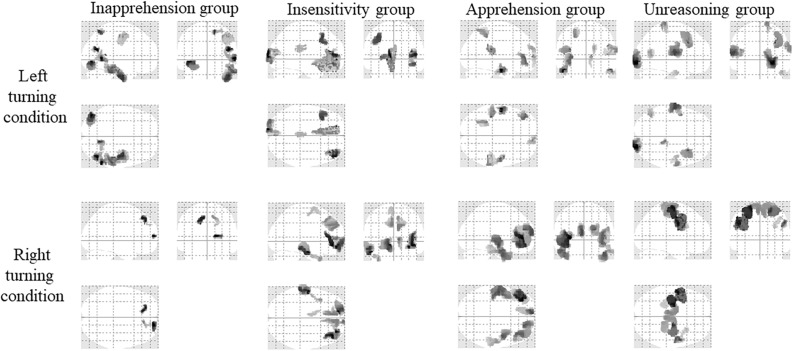
Activation of the four groups under the two conditions (SPM12, ANOVA, *P* < 0.05, FWE-corrected, extent threshold *k* > 70). The names of the groups are shown in the upside. The driving conditions are shown in the left side.

The EEG source reconstruction results of the four groups are shown in Figure 6 and the details are listed in Table 5. When turning left, in the Inapprehension group, the left inferior occipital gyrus, and right middle temporal gyrus, inferior temporal gyrus, precuneus, middle frontal gyrus and the precentral gyrus were activated; in he Insensitivity group, the left middle occipital gyrus, middle frontal gyrus, inferior frontal gyrus, calcarine and right middle frontal gyrus and the inferior frontal gyrus were activated; in the Apprehension group, the left superior parietal gyrus, middle temporal gyrus, middle frontal gyrus, and right superior frontal gyrus, supramarginal gyrus and the middle temporal gyrus were activated; in the Unreasoning group, the left postcentral gyrus, superior temporal gyrus, middle temporal gyrus, rolandic operculum, and right precentral gyrus, inferior occipital gyrus, calcarine, middle frontal gyrus and the postcentral gyrus were activated.

**TABLE 5:** Activation of the four groups under the left turning and right turning conditions.

							**Cluster size**
**Task**	**Group**	**Anatomy**	**Peak location**			***t***	**(Voxels)**
			***x***	***y***	***z***		
Turning left	Inapprehension group	Middle temporal gyrus	46	−68	20	6.46	468
		Inferior occipital gyrus	−42	−80	−6	6.33	572
		Inferior temporal gyrus	46	−16	−36	5.81	771
		Precuneus	14	−60	60	5.81	623
		Middle frontal gyrus	46	0	54	4.89	75
		Precentral gyrus	56	−2	46	4.67	134
	Insensitivity group	Middle occipital gyrus	−12	−102	8	5.70	639
		Middle frontal gyrus	46	48	6	5.47	383
		Middle frontal gyrus	−36	20	46	5.26	380
		Inferior frontal gyrus, orbital part	42	44	−12	5.16	305
		Calcarine	−8	−102	−2	5.03	314
		Middle frontal gyrus, orbital part	−2	54	−4	4.85	663
	Apprehension group	Middle temporal gyrus	−54	−10	−26	6.40	354
		Middle temporal	52	−14	−24	5.55	214
		Parietal operculum	−38	−32	18	5.52	323
		Middle frontal gyrus	−34	40	2	5.43	439
		Superior frontal gyrus, medial part	12	60	4	5.20	284
		Supramarginal gyrus	46	−40	26	5.15	489
		Superior parietal gyrus	−28	−44	48	5.15	435
	Unreasoning group	Inferior occipital gyrus	26	−98	−8	6.84	610
		Calcarine	18	−104	0	6.04	548
		Postcentral gyrus	−60	−12	14	6.01	466
		Superior temporal gyrus	−60	−12	12	6.00	477
		Middle frontal gyrus	32	18	36	5.90	885
		Postcentral gyrus	12	−32	76	5.45	366
		Rolandic operculum	−64	−4	8	5.27	70
		Middle temporal gyrus	−44	−62	8	5.08	322
		Precentral gyrus	48	−6	−28	4.71	71
Turning right	Inapprehension group	Superior frontal gyrus	18	60	10	5.15	137
		Superior frontal gyrus	−12	36	48	5.02	148
		Superior frontal gyrus	12	38	48	4.63	80
	Insensitivity group	Middle temporal gyrus	−52	−14	−24	6.00	192
		Middle frontal gyrus, orbital part	32	52	−14	5.93	445
		Superior frontal gyrus, orbital part	−12	56	−8	5.81	300
		Inferior frontal gyrus, triangle part	40	36	8	5.67	404
		Inferior temporal gyrus	−60	−30	−18	5.54	607
		Superior frontal gyrus, medial part	−6	44	34	5.00	550
		Superior frontal gyrus, medial part	12	54	32	5.00	621
		Superior frontal gyrus	16	52	32	4.95	81
		Supplementary motor area	−4	−8	58	4.59	190
	Apprehension group	Inferior frontal gyrus, triangle part	−34	40	4	8.07	649
		Middle frontal gyrus, orbital part	−38	44	−4	7.39	750
		Inferior frontal gyrus, triangle part	−40	32	10	7.35	927
		Superior frontal gyrus, medial part	−6	64	14	6.73	988
		Middle temporal	−46	−20	−4	6.49	333
		Middle frontal	44	40	6	6.41	700
		Inferior frontal gyrus, triangle part	40	34	10	6.38	569
		Middle frontal gyrus, orbital part	40	44	−6	6.36	461
		Superior frontal gyrus	16	52	22	6.05	142
	Unreasoning group	Postcentral gyrus	−54	−6	46	7.09	916
		Precentral gyrus	−50	−4	32	6.83	350
		Precentral gyrus	−24	−14	68	6.83	816
		Superior frontal gyrus	22	−12	62	6.30	561
		Precentral gyrus	52	0	36	6.14	716
		Paracentral lobule	−6	−24	60	5.41	242
		Paracentral lobule	4	−30	58	5.31	576
		Supplementary motor area	8	−12	68	5.19	75

*SPM12, ANOVA, *P* < 0.05, FWE-corrected, extent threshold *k* > 70. The location is in MNI coordinates.*

When turning right, in the Inapprehension group, the left and right superior frontal gyrus were activated; in the Insensitivity group, the left middle and inferior temporal gyrus, superior frontal gyrus, supplementary motor area, and right middle, inferior and superior frontal gyrus were activated; in the Apprehension group, the left and right inferior, middle and superior frontal gyrus, and the left middle temporal gyrus were activated; in the Unreasoning group, the left postcentral gyrus, paracentral gyrus, precentral gyrus, and right superior frontal gyrus, supplementary motor area, paracentral gyrus, and the precentral gyrus were activated.

#### Intra- and Inter-Group Comparison of EEG Source Reconstruction Results

An Intra-group comparison of the EEG source reconstruction indicated that there was a right turning > left turning activation difference in the left precentral gyrus (peak voxel at [−36 −8 50], *t* = 5.14, 479 voxels) in the Unreasoning group. There was no other intra-group activation difference between the two conditions.

**FIGURE 7:**
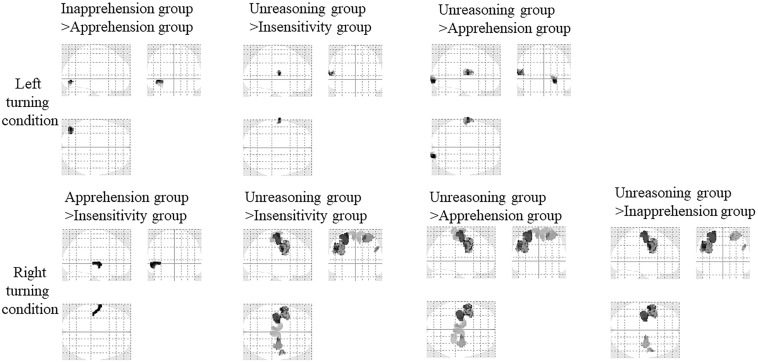
Activation comparison of the four groups under the two conditions (SPM12, ANOVA, *P* < 0.05, FWE-corrected, extent threshold *k* > 70). The driving conditions are shown in left side.

Results of the inter-group comparison are shown in Figure 7 and the details are listed in Table 6. Under the left turning condition, the Inapprehension group had stronger activity in the left inferior occipital gyrus compared to the Apprehension group. The Unreasoning group had stronger activity in the left superior temporal gyrus compared to the Insensitivity group, and in the right occipital pole and left central operculum compared to the Apprehension group.

**TABLE 6:** Activation comparison among the four groups.

							**Cluster**
**Task**	**Inter-group comparison**	**Anatomy**	**Peak location**			***t***	**size (Voxels)**
			***x***	***y***	***z***		
Turning left	Inapprehension group > Apprehension group	Inferior occipital gyrus	−40	−80	−6	4.97	220
	Unreasoning group > Insensitivity group	Superior temporal gyrus	−66	−12	10	4.68	96
	Unreasoning group > Apprehension group	Inferior occipital gyrus	24	−96	−8	5.24	256
		Superior temporal gyrus	−66	−12	10	5.12	336
Turning right	Unreasoning group > Insensitivity group	Postcentral gyrus	−54	−6	46	6.11	555
		Precentral gyrus	−52	−6	34	5.94	981
		Superior frontal gyrus	34	−6	62	4.92	71
		Paracentral lobule	−6	−24	60	4.75	388
		Supplementary motor area	4	−30	56	4.67	379
	Unreasoning group > Apprehension group	Postcentral gyrus	−54	−6	46	6.65	618
		Precentral gyrus	−34	−8	48	6.18	1075
		Paracentral lobule	−8	−24	60	4.97	497
		Superior frontal gyrus	22	−12	62	5.40	548
		Precentral gyrus	34	−24	68	4.88	252
		Paracentral lobule	6	−32	54	4.86	465
	Apprehension group > Insensitivity group	Superior temporal gyrus	−56	−8	−2	4.71	224
	Unreasoning group > Inapprehension group	Postcentral gyrus	−54	−6	46	6.20	595
		Precentral gyrus	−24	−14	66	5.68	982

*SPM12, ANOVA, *P* < 0.05, FWE-corrected, extent threshold *k* > 70. The location is in MNI coordinates.*

Under the right turning condition, the Unreasoning group had stronger activity in the left postcentral gyrus, precentral gyrus, paracentral lobule, and right precentral gyrus, superior frontal gyrus, and the supplementary motor area compared to the Insensitivity group, and in the left postcentral gyrus, precentral gyrus, paracentral lobule, and right superior frontal gyrus, precentral gyrus and the paracentral lobule compared to the Apprehension group, and in the left postcentral and postcentral gyrus compared to the Inapprehension group, the Apprehension group had stronger activity in the left superior temporal gyrus compared to the Insensitivity group.

## Discussion

In this study, 36 healthy subjects participated in a simulated driving experiment. The 64-channel EEG data and the driving data, including the real-time position of the vehicle, the rotation angle of the steering wheel and the speed were acquired simultaneously during driving. Through hierarchical clustering of the 16PF personality traits, the subjects were divided into four groups, i.e., the Inapprehension group, Insensitivity group, Apprehension group and the Unreasoning group, named after their representative personality trait. The driving data, the occurrence of aberrant driving behaviors and EEG source reconstruction results were compared between the four groups. The Unreasoning group had the highest occurrence of car collisions and the highest angular velocity during turning. For the subjects as a whole, the bilateral frontal and temporal gyrus were activated under the left turning and right turning conditions and no difference was detected between the two conditions. An intra-group comparison of the EEG source reconstruction indicated right turning > left turning activation in the left precentral gyrus in the Unreasoning group. An inter-group comparison indicated stronger activation of the temporal gyrus under the left turning condition and motor areas under the right turning condition in the Unreasoning group. Several other areas were also detected in the inter-group comparison, such as the inferior occipital gyrus (Inapprehension group > Apprehension group) and the superior temporal gyrus (Apprehension group > Insensitivity group).

### Correlation Between Personality and Driving

As shown in Tables 1–3, the number of car collisions were significantly different between four groups and were the highest in the Unreasoning group. The number of times driving out of the road were not significantly different between the four groups but were also the highest in the Unreasoning group. As for the performance in turning (Figure 4 and Table 3), the whole turn could be formulated into two steps, i.e., rotating the steering wheel toward the turning direction, modulating the head direction and entering the turn, and then rotating the steering wheel back and leaving the turn, which was in accordance with previous research (Xiong, 2010; Vesel, 2015). The Unreasoning group had the greatest absolute angular velocity in the two turning steps under the two driving conditions and the greatest rotation angle of the steering wheel in most circumstances (except in the second step of right turning). The total time of left turning of the Unreasoning group was the shortest, and second shortest in right (longer than Insensitivity group). Generally speaking, the greater rotation angle and higher angular velocity in turning corresponded to the more intensive modulation of the steering wheel, and were closely related with accidents (Vesel, 2015). These results indicated the worst driving performance and the most intensive driving action for the Unreasoning group. In the other three groups, the Inapprehension group had the lowest, but not significantly different, number of times of driving out of the road and there seemed to be no obvious difference in the turning performance between them.

People with a high Reasoning (B) score are intelligent, good at abstract thinking, and can learn quickly and correctly (Hilakivi et al., 1989; Manglam et al., 2013), while those with a low Reasoning (B) score are less intelligent, unable to handle abstract problems, think slowly and are suitable for trivial works (Hilakivi et al., 1989; Manglam et al., 2013). People with a high Sensitivity (I) score are sensitive, aesthetic, careful, dependent and lack confidence, while those with a low Sensitivity (I) score are utilitarian, objective, unsentimental, tough minded, careless, independent, realistic, decisive and confident, mature and are able to face reality (Zhang et al., 2009; Shi et al., 2017). People with a high Abstractedness (M) score are abstract, imaginative, absent minded, impractical, absorbed in ideas, imaginative, inattentive to things and careless, while those with a low Abstractedness (M) score are grounded, practical, prosaic, solution oriented, steady, conventional and serious (Zhang et al., 2009; Shi et al., 2017). People with a high Apprehension (O) score are apprehensive, self-doubting, worried, guilt prone and insecure, while those with a low Apprehension (O) score are confident, pretentious, smug and easily adapt to the environment (Brown, 1976; Hilakivi et al., 1989). People with a high Tension (Q4) score are tensive, highly energetic, impatient, driven, frustrated, over wrought, nervous, frustrated and often in a passive situation, while those with a low Tension (Q4) score are relaxed, placid, tranquil, torpid, patient, insensitive and sometimes unresponsive (Manglam et al., 2013; Yan, 2016). Previous 16PF research indicated that Social Boldness (H), Perfectionism (Q3), Dominance (E), Emotional Stability (C), Warmth (A) and Liveliness (F) were protective factors related to safe driving (Zhang et al., 2009; Sun, 2013; Yan, 2016; Shi et al., 2017), while Tension (Q4), Openness to Change (Q1) Abstractedness (M), Vigilance (L), Apprehension (O), Self-reliance (Q2), and Sensitivity (I) were risk factors related to dangerous driving behaviors (Suhr, 1953; Zhang et al., 2009; Shi et al., 2017).

The Unreasoning group had higher Tension (Q4) and Apprehension (O) scores and lower Reasoning (B) scores (Table 2), and were tense, highly energetic, impatient, less intelligent and were unable to handle abstract problems (Manglam et al., 2013; Yan, 2016). According to our results, together with the driving performance of the four groups, we speculated the positive correlation of Apprehension (O) and Tension (Q4) with dangerous driving and a negative correlation of Reasoning (B) with dangerous driving.

### Correlation Between EEG and Driving

We first analyzed the source reconstruction results of all the subjects. Under the left turning condition, the bilateral temporal gyrus, frontal and the occipital gyrus were activated. Under the right turning condition, the bilateral temporal gyrus and frontal gyrus were activated. No different activations were found between the two conditions. Then, the source reconstruction results of each group of subjects were analyzed and activation in the frontal gyrus was found in all groups. The temporal gyrus was detected in most groups and motor areas (precentral gyrus and postcentral gyrus) were strongly activated in the Unreasoning group. The occipital gyrus was activated in the Inapprehension group, Apprehension group and the Unreasoning group under the left turning condition. The activation of the Inapprehension group under right turning condition was restricted in the superior frontal gyrus.

To fulfill the turning behavior, the subjects needed to notice the turning sign, decide the turning direction and then to manipulate the steering wheel, which consisted of a series of visual-spatial and visual-motor processes. The brain regions related with vision, attention and motion, including the pre-supplementary motor area, the superior parietal and lateral occipital cortices and the cerebellum would be activated (Spiers and Maguire, 2007; Calhoun and Pearlson, 2012). The frontal gyrus was considered as an important area for visual attention (Corbetta and Shulman, 2002; Konen et al., 2004), decision-making (Volz et al., 2006; Glimcher et al., 2009), executive control (Christoff and Gabrieli, 2000; Koechlin and Summerfield, 2007; Posner et al., 2007), performance monitoring and adjustments (Ridderinkhof et al., 2004; Euston et al., 2012). The common activation of the bilateral frontal gyrus when turning left and right (Table 4 and Figures 5, 6) might be associated with these cognitive procedures. The occipital gyrus was activated in most groups only under the left turning condition. No significant difference was detected in the activations between the two turning conditions (FWE-corrected, *P* < 0.05, extent threshold *k* > 70). But if we applied a less conservative test (*P* < 0.01, uncorrected, extent threshold *k* > 70), left turning > right turning activation could be detected in the superior frontal (peak voxel at [−6 62 10], *t* = 3.14, 254 voxels; Supplementary Figure S1). As we described above, the frontal gyrus was involved in decision-making, executive control, performance monitoring and adjustments. The occipital gyrus played the important role in visual function (Lauritzen et al., 2009). Since motorists drive on the right-side in China, drivers are presumably accustomed to watching for traffic from both directions while turning left, which requires considerably stronger brain activity than with right turning (Schweizer et al., 2013; Oka et al., 2015). We speculated that the load of attention and visual information processing was more in left turning than right turning. It had been found that the superior temporal gyrus was an important structure in the pathway consisting of the prefrontal cortex and amygdala, which are all associated with social cognitive processes (Amanda et al., 2004; Callaghan et al., 2017). The stronger activation of the motor and sensorimotor areas in the Unreasoning group may relate with their more intensive movements, i.e., the greatest rotation angle and absolute angular velocity in turning (Tables 2, 3).

Some simulated driving studies investigated the underlying neural mechanisms of driving (Spiers and Maguire, 2007; Calhoun and Pearlson, 2012; Schweizer et al., 2013; Oka et al., 2015). The brain regions related with goal direction, attention and motor planning, including the frontal gyrus (Spiers and Maguire, 2007), the superior parietal cortex and lateral occipital cortex (Oka et al., 2015), pre-supplementary motor area and the cerebellum (Calhoun and Pearlson, 2012) were activated. The higher activation of bilateral parietal lobe were positively correlated with good driving performance (Uchiyama et al., 2012), while the activity of the anterior cingulate were negatively correlated with good driving performance and was involved in driving errors (Kan, 2011; Bledsoe et al., 2013). The inter-group comparison indicated that, under the left turning condition, the left superior temporal gyrus (Unreasoning group > Insensitivity group and Apprehension group) and right inferior occipital gyrus (Unreasoning group > Apprehension group) was detected (Figure 5 and Table 6). The superior temporal gyrus is an important area in the pathway consisting of the prefrontal cortex and amygdala, which are all associated with social cognitive processes (Amanda et al., 2004; Callaghan et al., 2017). The occipital gyrus is mainly involved in visual information processing (Lauritzen et al., 2009) and was found to be coupled with the parietal gyrus in sustained attention (Lauritzen et al., 2009) and spatial attention (Garg et al., 2007; Weaver and Stevens, 2007). The Unreasoning group had the greatest absolute angular velocity in the two turning steps under the two driving conditions and the greatest rotation angle of the steering wheel under most circumstances. The total time of left turning in the Unreasoning group was the shortest, and of right turning the second shortest (longer than the Insensitivity group). Their driving style seemed to be the most intensive and more easily made errors. To fulfill the same turning task, the time of the Unreasoning group was generally shorter than the other groups, which meant that they needed to process the same amount of information but in a shorter time. From this viewpoint, we think that the cognitive load of the Unreasoning group to process the turning information was higher.

The cognitive load could affect driving negatively, undermining drivers’ driving performance (Lee et al., 2007; Wijayanto et al., 2018). The increased cognitive load was associated with a common network comprising occipital cortices and parietal, thalamus, and the cerebellum (Tomasi et al., 2007). Among these areas, the occipital and parietal cortex are crucial in visual spatial attention functioning (Garg et al., 2007; Weaver and Stevens, 2007; Lauritzen et al., 2009). Visual spatial attention is a kind of attention, including a series of cognitive activities, such as visual searching, spatial area selection, attention switching and selective visual information processing in the useful field of view (Richardson and Marottoli, 2003; Wijayanto et al., 2018). Researches indicated that visual attention played an important role in predicting driving task performance, which is associated with a threefold increase in the risk of driving errors (Richardson and Marottoli, 2003). A higher load of visual spatial attention would diminish the sensitivity to the environment during driving and increase the risk of aberrant driving (Richardson and Marottoli, 2003; Lee et al., 2007), which is consistent with our results that the Unreasoning group are more likely to make errors and have poorer driving performance. Therefore, we speculated that the high occurrence of the aberrant driving behaviors and the intensive driving style in the Unreasoning group, were related with the higher load of visual spatial attention, when occipital areas played an important role.

Under the right turning condition, the Unreasoning group had stronger activity mainly in the bilateral postcentral gyrus, precentral gyrus and the paracentral gyrus compared to the other three groups (Figure 7 and Table 6). The stronger activation of these motor and sensorimotor areas may relate with the more intense movement of the Unreasoning group, i.e., the greatest rotation angle and absolute angular velocity in turning (Haseeb et al., 2007). Besides these areas, the superior frontal gyrus was also detected when comparing the Unreasoning group with the Insensitivity and Apprehension group. Considering the important role of the frontal gyrus in decision-making, executive control, performance monitoring and adjustments, its stronger activation here implied a higher load in these cognitive processes in the Unreasoning group compared to the other three groups. The Unreasoning group had the highest number of car collision with higher Apprehension (O) and Tension (Q4) scores and lower Reasoning (B) scores. We speculated that higher Apprehension (O) and Tension (Q4) and lower Reasoning (B) scores may cause dangerous driving and the superior frontal gyrus might play a very important role.

### Limitations of the Study

There are some limitations that should be considered in future studies. First, the samples were biased in gender, age and driving years. A previous study found that age (Callaghan et al., 2017), gender (Adenzato et al., 2017) and driving years (Pekkanen et al., 2018) were significant factors affecting a human’s cognitive and perceptive, decision making and spatial attention (Akamatsu et al., 2006). There were more male (75%) than female drivers in this study. The participant pool had relatively few and small personality differences. We compared the 16PF scores of the studied subjects and the national norm (Zhu and Dai, 1988) and found that the studied subjects had significantly different scores in Sensitivity (I), Abstractedness (M), Apprehension (O), perfectionism (Q3), Warmth (A), Dominance (E), Social Boldness (H), Vigilance (L), Privateness (N), and Openness to Change (Q1) (Supplementary Table S1). Second, the driving scenario was relatively complicated. The environment around the turns, and the parameters of the turns such as the radius and the length, were not exactly the same, which would affect the subjects’ reaction and brain activity to some extent. A simpler and more comparable scenario might be helpful in a quantitative analysis and comparison. Third, different to real driving, simulated driving cannot induce exactly the same experience and performance of the subjects since there was no real risk of a collision or actual injury. Under these circumstances, the underlying cognitive process and behavior may be distorted to some extent. Additionally, one subject failed to accomplish the driving tasks due to driving sickness. How to transplant the experiment and analysis schema safely and effectively to the real driving, is worth studying further. The ERPs utilized for resource reconstruction were acquired throughout the whole driving process, therefore, the effect of driving duration could not be detected using our current schema, which is another limitation of this study. Generally, driving duration had a close relationship with driving behaviors (Otmani et al., 2005; Geden et al., 2018) and EEG features (Puspasari et al., 2017). The influence of driving duration on personality, EEG and driving behaviors warrants further research.

Our study is currently, to some extent, an exploratory work. All the subjects were clustered into four groups based on their personality traits and then a *post hoc* comparison of their driving behaviors and EEG characteristics were conducted. We hoped to, and we did find a relationship between EEG, behavior and personality. If we could develop a large-scale study based on a larger sample size or if we could obtain the original data of the national norm, we might be able to extract all the typical and representative categories of the population, which can be applied as the standard and the new subjects could be classified based on this standard.

## Conclusion

In this paper, we explored the correlation between driving behavior, personality and EEG using a simulated driving experiment. The subjects were clustered into four groups, i.e., the Inapprehension group, Insensitivity group, Apprehension group and the Unreasoning group, according to their personality traits, using the hierarchical clustering method. The turning process of the subjects can be formulated into two steps, rotating the steering wheel toward the turning direction and entering the turn, and then rotating the steering wheel back and leaving the turn. The bilateral frontal gyrus was found to be activated when turning left and right which might be associated with its function in attention, decision-making and executive control functions in visual-spatial and visual-motor processes. The Unreasoning group had the worst driving performance with highest number of car collisions and the most intensive driving action, which was related to a higher load of visual spatial attention and decision making, when the occipital and superior frontal areas played a very important role. Apprehension (O) and Tension (Q4) had a positive correlation, and Reasoning (B) had a negative correlation with dangerous driving behaviors. Our results demonstrate the close correlation between driving behavior, personality and EEG.

## Ethics Statement

This study was carried out in accordance with the recommendations of the “Ethical Review Committee of the Wuhan University of Technology” with written informed consent from all subjects. All subjects gave written informed consent in accordance with the Declaration of Helsinki. The protocol was approved by the “Ethical Review Committee of the Wuhan University of Technology.” In this paper, we aimed to explore the relationship among personality traits, EEG and driving behavior, thus we needed to collect the electroencephalography signals of drivers during driving process. The whole experiment was completely harmless to the subjects.

## Author Contributions

FY and KG designed the data processing schema. YW, CD, and ML carried out the experiment. YW analyzed the data. LY and YW wrote the manuscript.

## Conflict of Interest Statement

The authors declare that the research was conducted in the absence of any commercial or financial relationships that could be construed as a potential conflict of interest.

## Footnotes

1 http://www.fil.ion.ucl.ac.uk/spm

## Supplementary Material

FIGURE S1 Activation of all subjects under left turning > right turning condition (SPM12, ANOVA, *p* < 0.01, uncorrected, extent threshold k > 70).
