# Supplementary figures and images for: Correlation Among Behavior, Personality, and Electroencephalography Revealed by a Simulated Driving Experiment
Source: Front Psychol. 2019 Jul 3;10:1524. doi: 10.3389/fpsyg.2019.01524 (PMC6626991; doi:10.3389/fpsyg.2019.01524)

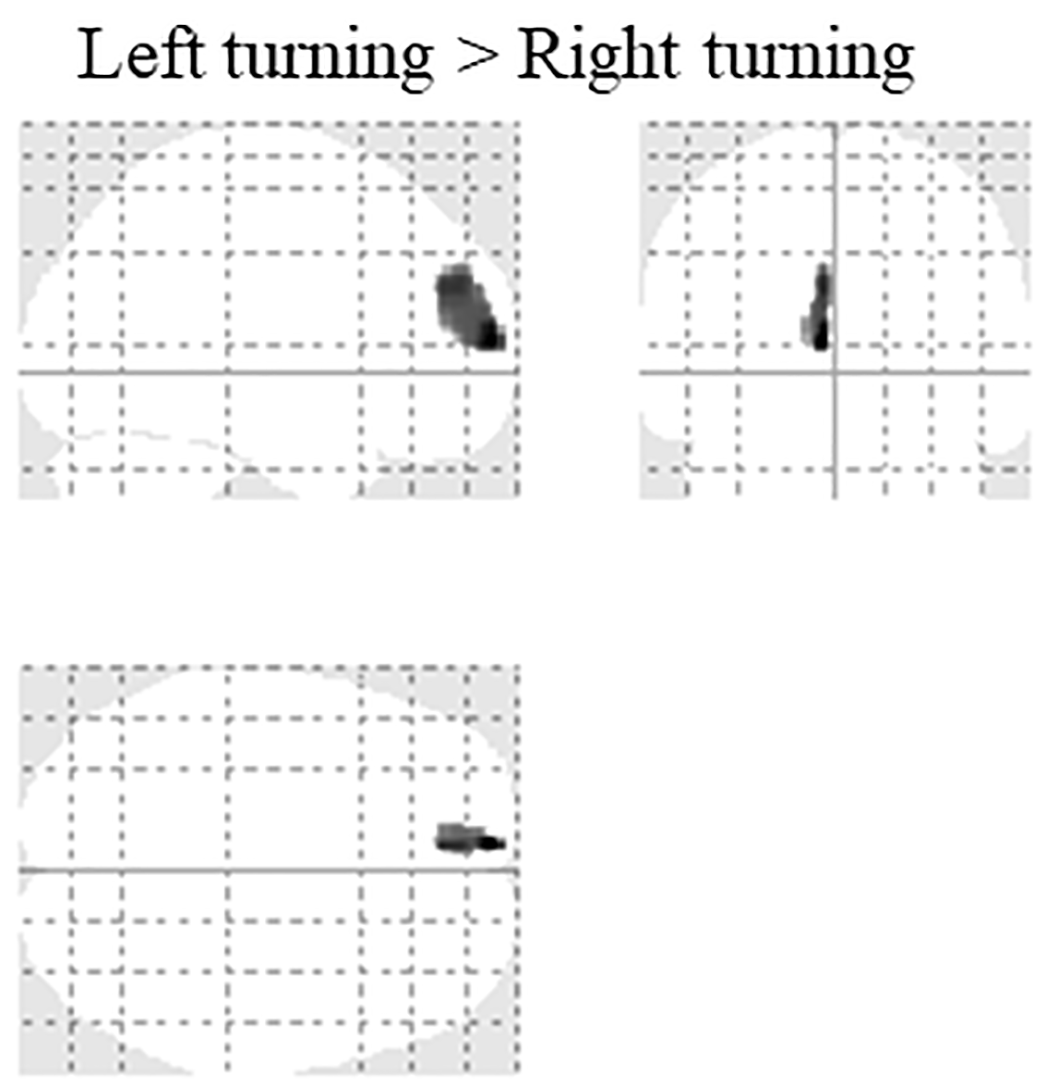

Supplement: FIGURE S1 — Activation of all subjects under left turning > right turning condition (SPM12, ANOVA, p < 0.01, uncorrected, extent threshold k > 70). [file Image_1.TIF]
