# Supplementary material for: Correlation Among Behavior, Personality, and Electroencephalography Revealed by a Simulated Driving Experiment
Source: Front Psychol. 2019 Jul 3;10:1524. doi: 10.3389/fpsyg.2019.01524 (PMC6626991; doi:10.3389/fpsyg.2019.01524)
Supplement: Supplementary file 2 [file Table_1.DOCX]

Table S1. Comparison of the original scores of the subjects and national norm **(**).

| Personality traits | male subjects (*n*=27) | male  norm | *t* | *P* | female subjects (*n*=9) | female  norm | *t* | *P* |
| --- | --- | --- | --- | --- | --- | --- | --- | --- |
| Warmth (A) | 8.44±3.81 | 10.02± 3.27 | -2.151^*^ | 0.041 | 9.22±3.74 | 10.90± 3.23 | -1.271 | 0.240 |
| Reasoning (B) | 9.04±2.56 | 8.65± 2.61 | 0.0784 | 0.440 | 9.22±1.75 | 8.90± 2.14 | 0.521 | 0.617 |
| Emotional Stability(C) | 14.56±3.33 | 15.00± 3.95 | -0.693 | 0.494 | 11.89±3.54 | 13.75± 3.96 | -1.486 | 0.176 |
| Dominance(E) | 10.37±2.65 | 12.77± 3.60 | -0.493^**^ | 0.000 | 8.89±2.28 | 11.70± 3.58 | -3.483^**^ | 0.008 |
| Liveliness(F) | 13.22±2.69 | 12.39± 4.40 | 1.605 | 0.121 | 12.67±5.66 | 12.84± 4.79 | -0.087 | 0.933 |
| Rule-Consciousness(G) | 12.07±3.16 | 12.63± 3.31 | -0.914 | 0.369 | 10.89±1.97 | 12.46± 3.14 | -2.257 | 0.054 |
| Social Boldness(H) | 9.85±2.93 | 11.07± 4.43 | -2.159^*^ | 0.040 | 9.00±3.46 | 10.47± 4.64 | -1.200 | 0.264 |
| Sensitivity(I) | 11.07±2.32 | 9.65± 2.95 | 3.191^**^ | 0.004 | 12.00±1.15 | 10.68± 2.91 | 3.233^*^ | 0.012 |
| Vigilance(L) | 8.26±2.31 | 10.95± 3.06 | -6.043^**^ | 0.000 | 9.67±1.41 | 10.23±3.36 | -1.127 | 0.293 |
| Abstractedness(M) | 13.85±2.54 | 10.89±3.51 | 6.065^**^ | 0.000 | 16.00±2.58 | 12.23±3.36 | 4.130^**^ | 0.003 |
| Privateness(N) | 7.70±2.09 | 9.66± 2.75 | -4.862^**^ | 0.000 | 8.11±2.33 | 9.94±2.95 | -2.219 | 0.057 |
| Apprehension(O) | 10.44±3.29 | 8.53± 3.64 | 3.028^**^ | 0.006 | 13.67±3.46 | 9.99± 3.84 | 3.002^*^ | 0.017 |
| Openness to Change(Q1) | 10.04±2.43 | 11.50± 2.91 | -3.134^*^ | 0.004 | 10.33±2.05 | 11.19± 2.90 | -1.179 | 0.272 |
| Self-Reliance(Q2) | 11.78±3.12 | 12.95± 3.34 | -1.954 | 0.062 | 11.33±2.87 | 11.65±3.21 | -0.312 | 0.763 |
| Perfectionism(Q3) | 13.26±2.71 | 12.16± 3.58 | 2.106^*^ | 0.045 | 13.22±1.93 | 12.24± 3.76 | 1.439 | 0.188 |
| Tension(Q4) | 11.26±3.17 | 10.78± 4.01 | 0.786 | 0.439 | 14.67±4.14 | 11.75± 4.21 | 1.994 | 0.081 |

Note. ^*^*P*<0.05; ^**^*P*<0.01.
